# OptiSyn: an interpretable, multi-omics–driven graph convolutional network framework for synergy-oriented drug combination design in disease treatment

**DOI:** 10.1186/s13020-026-01385-1

**Published:** 2026-03-24

**Authors:** Yinli Shi, Jun Liu, Guoduan Zeng, Yuedan Wang, Shuang Guan, Muzhi Li, Sicun Wang, Yanan Yu, Weibin Yang, Zhong Wang

**Affiliations:** 1 Institute of Basic Research in Clinical Medicine, China Academy of Chinese Medical Sciences, Beijing, China; 2 Quanzhou Orthopedic-traumatological Hospital, Fujian University of Chinese Medicine, Fujian, China; 3 Department of Gastroenterology, the Affiliated People’s Hospital of Fujian University of Traditional Chinese Medicine, Fuzhou, Fujian China; 4 Graduate School of China Academy of Chinese Medical Sciences, Beijing, China

**Keywords:** Interpretable modeling, Artificial intelligence, Multi-omics integration, Traditional Chinese medicine formula, Ankylosing spondylitis, T-cell immunity

## Abstract

**Background:**

Bioinformatics and large-scale computational modelling have emerged as essential research fields in modern biomedical science, enabling drug discovery and therapeutic optimisation. A unique and potent technical framework for the modernisation and mechanistic clarification of traditional Chinese medicine (TCM) formulations is provided by the integration of multidimensional data using systems biology and artificial intelligence (AI) techniques.

**Methods:**

Ankylosing spondylitis–associated key hub genes were identified using multi-omics datasets, differential gene expression analysis, weighted gene co-expression network analysis, single-cell transcriptomic analysis, Mendelian randomization, and network module partitioning. In order to predict the optimal drug combinations and synergistic principal-auxiliary therapeutic roles, an interpretable, multi-layer graph convolutional network model was built using network topology features, molecular docking data, empirical clinical medication knowledge, and compound clustering similarity.

**Results:**

Eight AS-associated hub genes were found using AS as a representative disease model. A possible TCM formula, ASD-A, comprising *Myrrha*, *Drynariae Rhizoma*, *Lycii Fructus*, *Epimedii Folium*, *Achyranthis Bidentatae Radix*, *Alpiniae Officinarum Rhizoma*, *Forsythiae Fructus*, *Astragali Radix,* was prioritised by the proposed model. While ablation studies highlighted the crucial role of multi-source information integration in compound formula screening and the creation of customised intervention methods, model performance evaluation showed strong predictive potential. The identified hub genes were found to be tightly linked to immunological responses and T-cell-mediated immune processes, according to functional enrichment analyses. Experiments conducted both in vivo and in vitro confirmed that ASD-A significantly reduced pro-inflammatory cytokines like IL-6 and TNF-α (*P* < 0.05), modulated the proportions of CD80 and CD86 cell subsets, and regulated the expression of important genes like KRAS, SMAD2, and MAPK14 (*P* < 0.05). Furthermore, in activated Jurkat cells, ASD-A significantly decreased IL17 fluorescence while increasing Foxp3 fluorescence (*P* < 0.05), indicating a rebalancing of the IL17/Foxp3 axis. The roles of major and auxiliary components in controlling hub gene activity were further clarified by formula decomposition analysis of ASD-A.

**Conclusion:**

The suggested AI-driven formula design approach provides new insights into combinatorial therapy approaches for AS while also conforming to the TCM theory of *Jun-Chen-Zuo-Shi* principle. The significant potential of combining contemporary bioinformatics and AI techniques with traditional medicine is demonstrated by this study, which could facilitate efficient and mechanistically informed disease therapy.

**Supplementary Information:**

The online version contains supplementary material available at 10.1186/s13020-026-01385-1.

## Introduction

The efficiency of medical diagnosis, predictive biomarker identification, and candidate drug discovery has significantly increased with the integration of multi-omics data, artificial intelligence (AI) techniques, and data-driven technologies [1, 2]. A growing emphasis on drug combinations and the intricacy of their interactions has gradually replaced traditional studies that only looked at dose–response relationships as a result of the expansion of pharmaceutical research and ongoing advancements in high-throughput screening technologies. Numerous computational techniques have been widely used to describe molecular structural features, clarify target gene functions, and improve the effectiveness of candidate drug prioritization [3]. These methods include machine learning, deep learning, virtual screening, pharmacophore modelling, and knowledge graph-based approaches [4]. In order to forecast drug combinations based on dose–effect relationships, these methods mainly rely on the analysis of massive amounts of raw data. Although they are frequently employed in Western medicine, there is growing evidence that these techniques can also accurately forecast the therapeutic results of interventions based on herbal medicine [5]. Combining systems biology, bioinformatics, chemical biology, transcriptomics, proteomics, metabolomics, and network pharmacology with genomics enables a comprehensive investigation of the intricate connections between disease, symptom patterns, molecular targets, and treatment recommendations. This approach is conceptually consistent with the holistic and syndrome-differentiation principles of traditional Chinese medicine (TCM) and makes it easier to understand the multifaceted synergistic and antagonistic mechanisms underlying herbal formulae [6]. When taken as a whole, this paradigm offers crucial support for AI-driven formula design while providing a theoretical and technical basis for the modernisation and precise implementation of conventional formulas [7].

Ankylosing spondylitis (AS) is a genetically predisposed chronic inflammatory rheumatic disease that mostly affects the sacroiliac joints and spine [8]. The pathophysiology of AS is still uncertain despite numerous studies, and it is believed to include intricate interactions between genetic, immunological, and environmental variables. There is growing evidence that T cells in AS patients have functional abnormalities, including a thrown-off balance between effector and regulatory T cells, which results in immunological intolerance and chronic inflammation [9]. As first-line treatments for AS, non-steroidal anti-inflammatory drugs can reduce pain and stiffness, but they frequently have less than ideal effects for controlling disease activity and delaying development [10]. Long-term management of biologic medicines must address adverse events and toxicity notwithstanding their great specificity and tailored efficacy [11]. Therefore, there is an urgent need to create new therapeutic approaches. Nevertheless, how to systematically integrate traditional formulas with modern multi-omics and AI-based approaches to enable rational screening and optimization of herbal combinations remains a critical unresolved challenge.

**Fig. 1:**
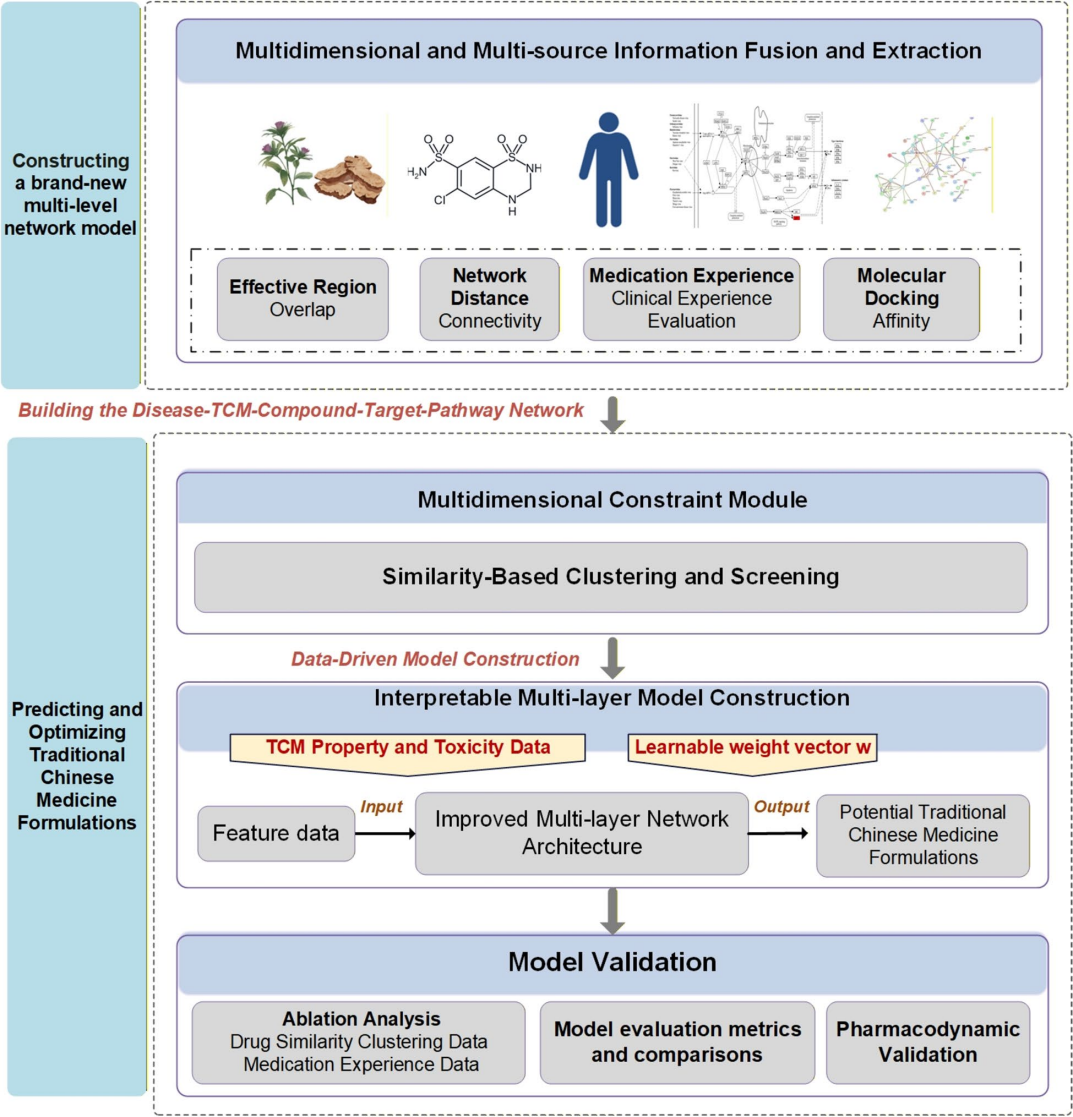
Schematic overview of the OptiSyn model experimental pipeline

An AI-driven framework that merges bioinformatics, computer-aided medication design, and current study evidence to efficiently screen disease-targeted drug combinations addresses this gap. In contrast to earlier methods, this framework not only helps modernise TCM formulas but also offers mechanistic insights into how different drug combinations interact, which makes it easier to move therapeutic strategies towards increased accuracy and system-level optimization [12]. The OptiSyn model's comprehensive methodology is presented in this section. We created an interpretable graph convolutional network-based synergy prediction model by building on our earlier work and using the idea of combining TCM theory with multi-target network modelling and deep learning technologies. This model functions on a multi-layer heterogeneous network that includes biological pathways, chemical substances, molecular targets, herbal remedies, and disease. Automated formula screening and synergistic potential prediction are made possible by the incorporation of key TCM qualities, such as the four properties and five flavours, meridian tropism**,** bioavailability, and drug-likeness criteria. Probability vectors for potential herbs and associated synergy scores are then obtained by using AS as a proof-of-concept application for herbal recommendation and synergistic combination prediction. Lastly, the general architecture and technological specifics of the suggested OptiSyn framework are thoroughly explained, and the model-generated formulas are validated pharmacodynamically both in vitro and in vivo.medications, chemical substances, molecular targets, and biological processes (Fig. 1).

## Materials and methods

### Data sources

Data on TCM compounds and related pharmacological information were obtained from the Traditional Chinese Medicine Systems Pharmacology Database (http://lsp.nwu.edu.cn/tcmsp.php) and DrugBank (https://go.drugbank.com/). All herb names were standardised according to the official nomenclature in the Chinese Pharmacopoeia and presented consistently throughout the manuscript using one unified format. All identified putative targets were standardized and deduplicated using the UniProt database (http://www.uniprot.org/). Clinical application and empirical usage information for TCM formulations were extracted from the “Chinese Patent Medicine Value Evaluation Information” database (http://crds.release.daodikeji.com) and the China National Knowledge Infrastructure (https://www.cnki.net/).Three-dimensional chemical structures of active compounds were retrieved from the PubChem database (https://pubchem.ncbi.nlm.nih.gov/), while corresponding protein receptor structures were obtained from the AlphaFold Protein Structure Database (https://alphafold.ebi.ac.uk/). Disease-related transcriptomic datasets were collected from the NCBI Gene Expression Omnibus (https://www.ncbi.nlm.nih.gov/geo/) using the keyword “ankylosing spondylitis”. The included gene expression datasets comprised PRJNA749866, GSE25101, GSE221786, and GSE11886. Using publically accessible resources, summary-level expression quantitative trait locus data for the disease were acquired (https://yanglab.westlake.edu.cn/software/smr/#eQTLsummarydata).

### Construction of a multi-layer heterogeneous “disease–herb–compound–target–pathway” network

#### *Identification of core disease targets *via* modular pharmacology*

The main molecular characteristics of AS were methodically described by integrating multi-level transcriptome data. First, the GSE221786 dataset was used to conduct a differential gene expression analysis. The sva program was used to eliminate batch effects, and the limma package was used to identify differentially expressed genes (DEGs) based on the thresholds of *P* < 0.05 and |log₂ fold change|> 1. ggplot2 was used to demonstrate the obtained DEGs. The GSE25101 dataset was then subjected to weighted gene co-expression network analysis (WGCNA). Hierarchical clustering was used to eliminate outlier data, and a suitable soft-thresholding power (R^2^ > 0.9) was used in order to build a network that was roughly scale-free. The topological overlap matrix and the dynamic tree cut technique were used to identify and combine gene modules. All computational techniques on the modular pharmacology analysis platform (http://112.86.129.72:48081) were used to filter core modules using module preservation analysis and multi-criteria ranking [13, 14]. PRJNA749866 single-cell RNA sequencing data was evaluated using Seurat for quality control, normalization, principal component analysis, and cellular gene refinement. Marker genes from the CellMarker (http://xteam.xbio.top/CellMarker/) database were used to annotate cell types. In parallel, summary-level expression quantitative trait locus data from GTEx v8 were integrated using summary-data-based Mendelian randomization (SMR) to investigate genetic variant-gene expression relationships in AS. Pleiotropy and linkage effects were distinguished using the HEIDI test. Genes with SMR *P* < 0.05 and HEIDI *P* > 0.05 were kept. A random walk technique was used to assess gene relevance in the network, and candidate genes were prioritized using a PageRank threshold of 3.00 × 10⁻^3^. STRING database (https://string-db.org/) was used to create protein–protein interaction (PPI) networks from differential expression analysis, WGCNA, and scRNA-seq results. Molecular complex detection, Markov clustering, and GLay discovered functional modules in each PPI network. A composite evaluation approach was then used to score and rank each module [15].

#### Target-herb initial association assessment based on overlap and effectiveness scores

To help figure out if there might be a therapeutic link between certain herbal medicines and the disease, the overlap rate was used to measure how closely the target set of a single herb overlapped with the disease-associated target set [16]. Additionally, the Hscore, an integrated scoring measure, was created to provide more information about how well the herbs work in the disease network. A multi-layer herb–compound–disease network employing PageRank and a two-step random walk method was used to calculate the Hscore, which captured herbal active compounds' worldwide regulatory potential on disease-related targets. Herbs with more bioactive compounds have a wider range of targets and a bigger effect on networks.

#### Network proximity analysis within the disease-protein interaction module

Genes linked with certain herbal remedies tend to form coherent modules within the PPI network, and their network proximity to diseases-associated genes demonstrates the functional similarity between herbal targets and disease mechanisms [17]. The network relationship between disease targets and drug targets, denoted as the mean shortest distance (S_AB_), was quantified by comparing the average shortest path length within each target set to the average shortest path length between the mean shortest distance (d_AB_). The related Z-score is a reliable indicator of network proximity between herbal targets (*X*) and disease-associated genes (*Y*), allowing for a quantitative assessment of their potential therapeutic value.

#### Clinical co-usage profiling and empirical herb association scoring

A method for evaluating pharmacopeias based on clinical experience was created to quantitatively describe the patterns of herbal co-administration in real-life clinical practice. The Chinese Patent Medicine Value Evaluation Information database was used to find and organize a total of 28,279 plant pair combinations. For each herbal pair, the Pscore was determined after it was systematically retrieved, deduplicated, and standardized. Pscore shows the empirical combination patterns that have been formed over many years of clinical practice [18]. It is a measure based on experience of how well herbal mixtures might work. To make the pharmacopeia analysis more useful and clinically relevant, all published TCM formula studies about the target disease from January 2019 to December 2024 were gathered from the CNKI. After deduplication and normalization, factor analysis was performed on herbal pair and formula datasets to evaluate herb frequencies in high-occurrence herbal combinations. The Mscore reflects a herb's representativeness and relevance in the therapeutic co-drug system by measuring its average co-occurrence frequency across regularly utilized herbal pairs.

#### Molecular docking-based identification of representative compounds

It was decided which compounds with a frequency of occurrence of 5 or more would be used for molecular docking research against genes linked to disease. By eliminating water molecules and co-crystallized ligands, receptor protein structures were created using PyMOL (version 2.6). AutoDock Vina was used to run molecular docking simulations, and each ligand-receptor pair's binding affinities were determined.

#### Structure-based similarity clustering for key compound selection

Molecular fingerprints of the representative compounds were generated using the rcdk, ChemmineR, and cluster packages. Molecular fingerprint similarity was quantified using the Tanimoto coefficient. K-means clustering, a widely adopted algorithm in drug discovery, was then applied to group compounds within each herb into three clusters based on their pairwise distance measures. To assess the robustness and validity of the clustering results, the Dunn index and the silhouette score were employed as evaluation metrics, with an average silhouette score of 0.5 used as the acceptance threshold [19, 20]. Based on these results, a multi-layer “disease-herb-compound-target-pathway” network was constructed. The key parameters and formula definitions required for network construction are summarized in Table 1.

**Table 1:** Definitions of indicators and mathematical formulations for multilayer heterogeneous network construction

Role	Metric	Equation	Explanation
Target-Herb Initial Association Assessment	Overlap rate		where nHerbi​​ represents the gene set size of the i-th herb, nHub-Dis​ denotes the size of the disease gene set, and ∣nHerbi​​ ∩ nHub-Dis​∣ refers to the cardinality of the intersection between the two gene sets
	Hscore		where Aβ​ denotes the targeting score of β; Vβγ​ is the adjacency matrix element between nodes β and γ, taking values of 1 or 0 to indicate whether an edge exists; and βout denotes the out-degree of node β within the network
Analysis of Proximity and Separability in Modular Network Topological Structures	*s*_*AB*_		where dAA​ and dBB​ represent the average shortest distances within the respective interaction neighborhoods of node sets A and B. A negative sAB​ indicates topological overlap between the two target sets, whereas a non-negative sAB​ suggests topological separation
	*d*_*AB*_		Where dAA​ and dBB​ denote the mean shortest path lengths within the interaction neighborhoods of node sets A and B, respectively. When sAB < 0, the two target sets exhibit topological overlap, whereas when sAB ≥ 0, they are considered topologically separated. The mean and standard deviation are denoted by μ and σ, respectively. From a network-based perspective, if the targets of the Chinese herbal medicine are topologically separated from AS–associated proteins, the corresponding z ≥ 0; otherwise, z < 0
Discovery of Drug Association Patterns from Real-World Medication Experience	Pscore		Where Herb*i* and Herb*j* both denote Chinese herbal medicines;Pscore*j* represents the sum of Pscores of all herb pairs retrieved from the database that contain a given herb Herb*j*;*n* denotes the number of such retrieved herb pairs;and ε denotes the maximum absolute factor loading of a given herb Herb*i* obtained from factor analysis
	Mscore		
Collaborative Filtering–Driven Similarity Estimation with Clustering Validity Evaluation	Dunn idex		Where dii denotes the shortest distance between different clusters, and dkl represents the maximum distance within the same cluster
	Silhouette score		Where a(i) denotes the average dissimilarity between vector i and all other points within the same cluster, and b(i) represents the minimum average dissimilarity between vector i and points in any other cluster

### Graph convolutional network-based herb synergy prediction with singular value decomposition-optimized formulas

To model potential synergistic interactions among herbs, we constructed a graph convolutional network (GCN)—based framework that integrates biological features, pharmacological attributes, and network topology information. In this framework, herbs were represented as nodes in a graph, and potential compatibility relationships between herb pairs were modeled as edges. The learning task was formulated as a supervised edge-level classification problem, in which the model predicts whether a pair of herbs forms a potential synergistic combination. Binary labels were defined as the supervision signal for model training. Positive samples (label = 1) consisted of herb pairs that co-occur in TCM prescriptions or have been reported as compatible combinations in the literature. Negative samples (label = 0) were generated by randomly sampling herb pairs from the candidate herb set that do not co-occur in known prescriptions or reported compatibility records. These labels were derived from curated prescription databases and literature sources. Each herb pair therefore represents an edge-level training instance with a binary classification label.

The herb interaction network was represented as a graph $G=(V,E)$, where $V$ denotes the set of herb nodes and $E$ represents candidate herb–herb interactions. Each herb node was associated with a feature vector describing its biological and pharmacological characteristics derived from herb–target–disease associations and network topology metrics. These features included network distance measures (d_ab_, S_ab_, and max.diameter), chemical and pharmacological indicators (the average compound effectiveness number (Ec), overlap rate, Hscore, Mscore, and average silhouette score), drug–drug associations, drug–disease associations, compound docking capability, and compound efficacy scores. It is important to note that Ec represents a model-derived score reflecting the potential association of compounds with drugs and disease-related targets. Node features were organized into a matrix $X\in {\mathbb{R}}^{N\times F}$, where $N$ denotes the number of herb nodes and $F$ represents feature dimensionality. All global network features and node attributes were computed prior to dataset splitting to ensure consistency and to avoid potential information leakage during model training.

To estimate herb compatibility, the interaction score of each herb pair was calculated as the inner product of their learned node embeddings. The resultant score was then converted to a probability using a sigmoid activation function, indicating the possibility of a synergistic interaction. The model parameters were tuned by reducing the binary cross-entropy loss between predicted probability and ground-truth herb pair labels. The detailed mathematical formulations are provided below:

1 $${E}_{C}=\frac{1}{{N}_{Herb}}\bullet \frac{{n}_{Herb({j}^{Rc})}}{{n}_{Herb(j)}}$$

2 $${H}^{(l+1)}=(A{H}^{(l)}\bullet {G}^{(l)})$$

3 $${Score}_{(Herb(i),Herb(j))}={h}_{Herb(i)}^{T}\bullet diag(w)\bullet {h}_{Herb(j)}$$

4 $${Prob}_{(Herb(i),Herb(j))}=\upsigma ({Score}_{(Herb(i),Herb(j))})=\frac{1}{1+{e}^{-score(Herb(i),Herb(j))}}$$

5 $$\widehat{y}(Herb(i),Herb(j))=\left\{\begin{array}{c}1, if{Prob}_{(Herb(i),Herb(j))}>0.5\\ 0, otherwise\end{array}\right.$$

In the equations, $N$ denotes the number of core herbs, ${N}_{Herb}$ represents the total number of effective compounds identified through molecular docking, ${n}_{Herb(j)}$ corresponds to the total number of compounds contained in herb ${Herb}_{j}$, and ${n}_{Herb({j}^{Rc})}$ represents the number of effective compounds within herb ${Herb}_{j}$. $A$ denotes the normalized adjacency matrix of the graph, and ${G}^{\left(l\right)}$ represents the weight matrix at the $l$-th layer of the GCN.

Prior to model training, key hyperparameters were systematically tuned based on validation performance, including hidden layer dimensions (16–128), learning rate (1 × 10⁻^2^–1 × 10⁻^5^), dropout rate (0–0.6), and number of training epochs. Hyperparameter optimization was conducted using a grid search strategy combined with early stopping to balance model performance and generalization ability. The final hyperparameter configuration used in this study is summarized in Table 2. In addition, singular value decomposition (SVD) was applied to normalized feature matrices to extract principal components and reduce feature redundancy [21]. Pairwise herb correlation coefficients were subsequently calculated using vector inner products, allowing the integration of herb intrinsic properties with their regulatory relationships to disease and compound nodes. Based on these SVD-derived correlations, herbs predicted by the GCN model were further ranked using the GCN Degree and Average Drug Association scores. For model evaluation, the dataset was randomly divided into training and testing sets at a 70:30 ratio, and performance was further assessed using threefold cross-validation. To ensure robustness and reduce randomness effects, all experiments were repeated with multiple random seeds, and the final results were reported as the average performance across runs. The model was implemented using the PyTorch frameworks.

**Table 2:** Final hyperparameter settings of the GCN model

Hyperparameter	Search space/description	Final setting
Hidden dimension	16,32,64,128	32
Learning rate	1 × 10⁻^2^, 1 × 10⁻^3^, 1 × 10⁻^4^,1 × 10⁻^5^	1 × 10⁻^4^
Dropout rate	0,0.1,0.2,0.3,0.4,0.5,0.6	0.5
Maximum epochs	50,100,200,300	100
Optimizer	Adam	Adam
Loss function	Binary cross-entropy	Binary cross-entropy
Gradient clipping	Applied for training stability	Max norm = 1.0

### Benchmarking against mainstream machine-learning models

Model performance was compared with seven standard machine learning algorithms: decision tree (DT), random forest (RF), gradient boosting decision tree (GBDT), support vector machine (SVM), extreme gradient boosting (XGBoost), LightGBM, and k-nearest neighbors (KNN). Key metrics included accuracy, precision, recall, F1 score, and AUC for classification, as well as RMSE and MAE for regression.

### Ablation study on compound clustering and clinical experience contributions

Ablation analysis was performed to evaluate the contributions of compound similarity clustering and clinical experience data. The full OptiSyn model generated formula A (ASD-A), while sequential exclusion of compound clustering (OptiSyn-S) and clinical usage information (OptiSyn-E) yielded formulas B (ASD-B) and C (ASD-C), respectively. Resulting changes in regression and classification metrics were compared to quantify the effect of each component.

### Biological and clinical validation of hub targets

With R packages, GO and KEGG enrichment studies of hub genes were done, and bubble and Sankey plots were used to show the results (P < 0.05, q < 0.05). CIBERSORT was used to measure immune infiltration, ssGSEA was used to measure immune-related processes, and Spearman correlation was used to measure connections with hub genes. The independent GEO dataset GSE11886 was used to validate core target expression, and ROC curves with an AUC > 0.5 were used to evaluate diagnostic performance.

### In vivo and in vitro validation

#### Animal modeling and treatment

Ninety-six SKG mice (ZAP-70 mutant autoimmune model) and twelve male SPF BALB/c mice (6–8 weeks old) were obtained from a germ-free breeding facility (Zhongyan Zichuang Biotechnology, Beijing, China). All procedures were approved by the Institutional Animal Care and Use Committee (approval no. ZYZC202508007S). Curldan (3 mg/mouse, once weekly for 2 weeks) was injected intraperitoneally into SKG mice to cause disease, while BALB/c controls were given saline [22]. A total of six groups were made up of successfully modeled SKG mice: model (AS), methotrexate (MTX), betulinic acid (BA), and herbal recipes ASD A-C. Herbal formulations were given orally (1 g/kg/day), MTX intraperitoneally (1 mg/kg every 3 days), and BA intraperitoneally (5 mg/kg/day) for 4 weeks [23, 24].

#### Clinical and histological assessment

Arthritis severity was scored weekly in a double-blind manner, and paw thickness was measured with a caliper. At study endpoint, mice were euthanized, spinal tissues collected, fixed in 4% paraformaldehyde (Solarbio, Beijing), and stained with hematoxylin and eosin (H&E, Beyotime, Shanghai) to quantify lesion severity.

#### Micro-computed tomography (Micro-CT)

Micro-CT was used to image the animal's spine and hind paws in three dimensions. These were the scanning parameters: At 40 kV voltage, 500 μA current, and 40 ms exposure time, the image resolution is 768 × 972 pixels. To differentiate between mature and newly formed bone, segmentation thresholds were set based on bone mineral density. For the left hind paw, mature bone measures 800–3000 mg HA/cm^3^, while newly formed bone measures 400–750 mg HA/cm^3^. For the L5-L6 vertebrae, mature bone measures 550–3000 mg HA/cm^3^, and newly formed bone measures 200–500 mg HA/cm^3^ These criteria quantified bone development and remodeling.

#### Flow Cytometry Analysis

Fresh mouse blood was collected and adjusted to 1 × 10⁶ cells per tube, then incubated with anti-CD80 (batch: 46-0801-82, Thermo Fisher, USA) or anti-CD86 (batch: 17-0862-82, Thermo Fisher, USA) antibodies according to the manufacturer’s instructions. Samples were analyzed using an EPICS flow cytometer, and data were processed with FlowJo V10 software to quantify the ratios of CD80⁺ and CD86⁺ cells. The gating strategy involved sequential selection of live cells using the viability dye (FVD-eFluor 780), followed by gating on CD45⁺ cells. CD80⁺ and CD86⁺ cells were then quantified within the live CD45⁺ parent population. Positivity thresholds were defined with reference to appropriate single-stain/control samples, and fluorescence compensation was performed before analysis.

#### Cell line establishment and culture

Jurkat cells were obtained from Wuhan Pronose Life Science Co., Ltd. (batch: CL-0129, China) and cultured in RPMI-1640 medium supplemented with 10% fetal bovine serum and 1% penicillin–streptomycin at 37 °C under a gas mixture of 21% O₂, 5% CO₂, and 74% N₂. To simulate immune activation, cells were stimulated with 1 μg/mL anti-CD3 (batch: 11-0037-42, Thermo Fisher, USA) and 1 μg/mL anti-CD28 (batch: 16–0289-85, Thermo Fisher, USA) antibodies [25].

#### CCK-8 assay

Jurkat cells (5 × 10^4^ cells/mL) were seeded in 48-well plates and treated with graded concentrations of herbal formulas, MTX, or BA for 24 and 48 h. Following treatment, CCK-8 reagent (batch: AQ308, Beijing AoQing Biotechnology, China) was added and incubated at 37 °C for 2 h. Cell viability was assessed via OD measurement to determine optimal intervention concentrations.

#### Immunofluorescence staining

Once the stated stimulation was done, Jurkat cells were washed with PBS, fixed in 4% paraformaldehyde, and made permeable with 0.1% Triton X-100. After blocking the cells with 5% BSA, the primary antibody against IL17 (batch: sc-374218, Santa, USA) and Foxp3 (batch: sc-53876, Santa, USA) were incubated for the entire night at 4 °C. Cells were rinsed with PBS the next day, treated with a fluorescent secondary antibody, rinsed once more, and then counterstained with DAPI. Fluorescence imaging was used to look at how much IL17A and Foxp3 were expressed.

#### Enzyme-linked immunosorbent assay (ELISA)

Mouse serum was analyzed following the manufacturer’s instructions to determine levels of hematological parameters (red blood cells, white blood cells, hemoglobin; Nanjing Jiancheng, China), liver function markers (ALT, AST; Nanjing Jiancheng, China), kidney function markers (creatinine, blood urea nitrogen; Nanjing Jiancheng, China), and inflammatory cytokines TNF-α (batch: KE00154) and IL-6 (batch: KE00139; Proteintech, China). All measurements were normalized to total content.

#### RNA extraction and q-PCR

Total RNA from spinal tissues, surrounding muscles/ligaments, and Jurkat cells was extracted using a column-based kit (RC113, Vazyme, China). RNA quality was assessed spectrophotometrically. cDNA synthesis and q-PCR were performed per kit instructions (R423-01, Q712-02, Vazyme, China) using GAPDH as the internal control. PCR cycling: 95 °C for 30 s, 40 cycles of 95 °C for 10 s and 60 °C for 30 s. Relative mRNA levels of KLRK1, KLRC2, EOMES, ERK1, and ERK2 were calculated via the 2^⁻ΔΔCt^ method (primers in Table 3).

**Table 3:** Sequences of qPCR primers for human and mouse genes

Gene	Sequence (5'—> 3')	Length
Human-derived Jurkat cells		
KRAS	Forward Primer	ACAGAGAGTGGAGGATGCTTT
	Reverse Primer	TTTCACACAGCCAGGAGTCTT
MAPK14	Forward Primer	CCCGAGCGTTACCAGAACC
	Reverse Primer	TCGCATGAATGATGGACTGAAAT
SMAD2	Forward Primer	CGTCCATCTTGCCATTCACG
	Reverse Primer	CTCAAGCTCATCTAATCGTCCTG
GAPDH	Forward Primer	TCTTGCTCAGTGTCCTTGC
	Reverse Primer	CTTTGTCAAGCTCATTTCCTGG
Mouse-derived sequence		
KRAS	Forward Primer	GCTCAGGAGTTAGCAAGGAG
	Reverse Primer	GTATTCACATAACTGTACACCTTG
MAPK14	Forward Primer	TGACCCTTATGACCAGTCCTTT
	Reverse Primer	GTCAGGCTCTTCCACTCATCTAT
SMAD2	Forward Primer	ATGTCGTCCATCTTGCCATTC
	Reverse Primer	AACCGTCCTGTTTTCTTTAGCTT
ERK1	Forward Primer	GAGGCGGGAGGAGTGGAGATGGC
	Reverse Primer	GTTCTTGTTAGGGGCCCTCTGGC
ERK2	Forward Primer	CCAACCATTGAGCAAATGAA
	Reverse Primer	CAAGGCCAAAGTCACAGATC
KLRC2	Forward Primer	CTCATGGATTGGTGTGTTTCGT
	Reverse Primer	CACTGTAAACGCAAATGCTTTACTTC
KLRK1	Forward Primer	ACTCAGAGATGAGCAAATGCC
	Reverse Primer	CAGGTTGACTGGTAGTTAGTGC
EOMES	Forward Primer	TGAATGAACCTTCCAAGACTCAGA
	Reverse Primer	GGCTTGAGGCAAAGTGTTGACA
GAPDH	Forward Primer	CGACTTCAACAGCAACTCCCACTCTTCC
	Reverse Primer	TGGGTGGTCCAGGGTTTCTTACTCCTT

#### Western blot

Proteins from mice spinal tissues, surrounding muscles/ligaments, and cells were extracted in RIPA buffer with PMSF, denatured, and separated on 10% SDS-PAGE gels. Proteins were transferred to 0.45 μm PVDF membranes, blocked with 5% milk, and incubated overnight with primary antibodies against (batch: 83,841-4-RR, Proteintech, China, 1:5000), KRAS (batch: 12,063-1-AP, Proteintech, China, 1:5000), MAPK14 (batch: 66,234-1-Ig, Proteintech, China, 1:2000), and GAPDH (batch: 60,004-1-Ig, Proteintech, China, 1:50,000). After secondary antibody incubation (Proteintech, China, 1:3000), bands were detected via ECL chemiluminescence and quantified using ImageJ.

#### Deconstruction of the herbal formula

To assess the synergistic effectiveness of the AI-predicted herbal formula, the optimal formulation was analyzed for functional validation in animal studies. According to model-derived combination scores, the top 25% of herbs were categorized as core components (ASD-M), while the others were classed as auxiliary components (ASD-S). Both groups underwent the previously outlined induction and treatment regimens, facilitating a thorough evaluation of the functional contributions and synergistic interactions of individual components within the overall formula.

#### UPLC-QTOF-MS analysis

The optimal formulation was filtered through a 0.22 μm membrane and analyzed using a Waters ACQUITY UPLC™ system with a BEH C18 column (2.1 × 100 mm, 1.7 μm). The mobile phase consisted of 0.2% formic acid in water (A) and 0.1% acetonitrile (B), with a 1 μL injection at 300 μL/min. Gradient elution was 5–95% B over 0–30 min, then 95–5% B over 30–33 min. Mass spectrometry was performed in negative ESI MSe mode on a UPLC-QTOF-MS system. Key parameters: scan range m/z 50–1500, scan interval 0.2 s, capillary voltage 3.0 kV, cone voltage 40 V, desolvation gas 800L·h⁻^1^, source temperature 120 °C, desolvation temperature 450 °C, and cone gas flow 50 L·h⁻^1^ (N₂).

### Statistical analysis

All data were analyzed and plotted using GraphPad Prism 9.0. Values are presented as mean ± SEM. For normally distributed data with homogeneous variances, comparisons between two groups were performed using the Student’s t-test, while multiple-group comparisons were conducted using one-way ANOVA followed by LSD post hoc tests. A P-value < 0.05 was considered statistically significant.

## Results

### Identification of hub genes associated with AS

**Fig. 2:**
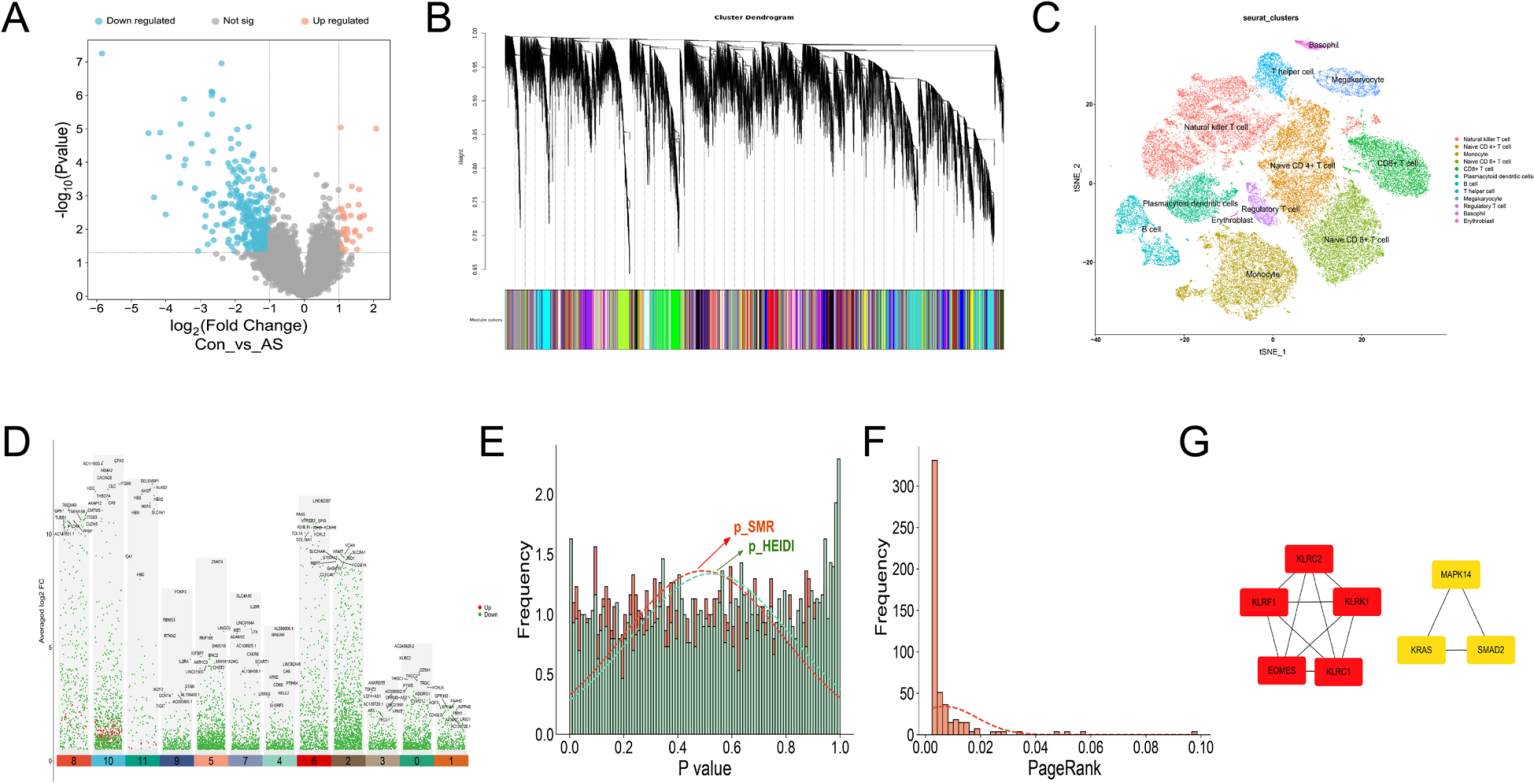
Identification of AS-associated hub genes. **A** Volcano plot of DEGs. **B** WGCNA showing the hierarchical clustering dendrogram, with distinct colors representing different modules. **C** Dimensionality reduction of single-cell transcriptomic data. **D** Distribution of key genes across cell clusters identified by single-cell analysis. **E** Gene density distribution derived from SMR analysis. **F** Gene prioritization based on the random-walk algorithm. **G** Construction of the PPI network and identification of hub genes

A comparison of samples from the AS group and healthy controls revealed 466 DEGs, comprising 410 downregulated and 56 upregulated genes (Fig. 2A). Hierarchical clustering, in conjunction with dynamic tree cutting, was utilized to identify and eliminate outlier samples. A topological overlap matrix was generated with a soft-thresholding power of 7, leading to the discovery of 139 gene modules by WGCNA (Fig. 2B). The quality assessment of RNA-seq data revealed robust transcriptome profiles, identifying 11 major cell types in AS, including NK cells, T cells, monocytes, and dendritic cells. A total of 3,901 genes were discovered using single-cell RNA sequencing analysis (Fig. 2C, D).

Following the integration and deduplication of differentially expressed genes, genes from significant WGCNA modules, and single-cell marker genes, a total of 4,348 potential genes linked with AS were identified. The SMR analysis, along with random-walk prioritization, found 58 genes with robust genetic correlations with AS, which were utilized to form a PPI network (Fig. 2E, F). Utilizing the minimal entropy concept, the MCODE algorithm was chosen to identify densely connected protein clusters inside the network (Fig. 2G). Clusters 1 and 2 demonstrated the highest scores and were consequently prioritized, resulting in the definitive identification of KLRF1, KLRC2, KLRK1, EOMES, KLRC1, SMAD2, KRAS, and MAPK14 as hub genes associated with AS.

### Multi-perspective filtering of herbal candidates targeting hub genes

**Fig. 3:**
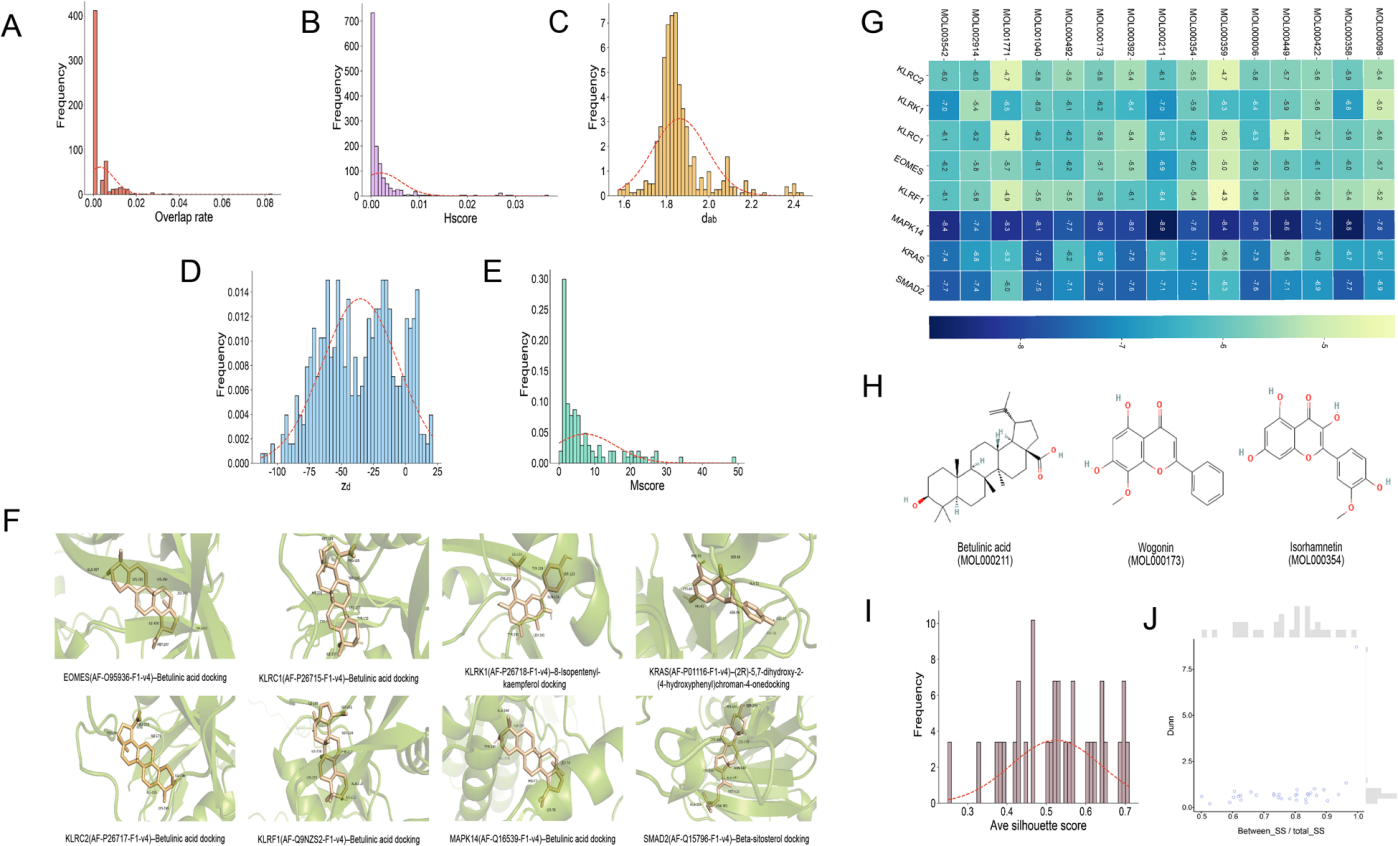
Multi-dimensional criteria for herb prioritization based on a multilayer network framework. **A** Overlap rate. **B** Hscore. **C** d_AB_. **D** Z_d_. **E** Mscore. **F** Hub gene-optimized compound molecular docking. **G** A heatmap summarizing docking affinity profiles. **H** High-frequency representative active compounds. **I** The average silhouette score. **J** Dunn index

After 473 herbs in the TCMSP database were filtered for oral bioavailability and drug-likeness, 455 herbs showed target overlap with hub genes linked to AS. Applying an overlap-based threshold retained 109 candidate herbs, whose target PPI networks were constructed and evaluated for network proximity to disease modules. The majority of herb targets showed significant topological intersections with hub genes (Fig. 3A, B). The Hscore measure (Overlap rate > 0 and Hscore > 0.001) was used to further rank the herbs, and network proximity analysis frequently showed negative Z-scores (Z < 0), indicating non-random topological closeness between herb targets and disease modules (Fig. 3C, D).

MTX, a first-line pharmacological agent for AS, was used as a reference drug (d_AB_ = 2.167, Z =  − 4.318), resulting in the selection of 105 herbs with comparable or stronger network proximity. The candidate group was further narrowed to 45 herbs with 612 chemicals by adding clinical usage frequency (Mscore > 5) (Fig. 3E). Molecular docking analysis revealed that 95% of ligand–target interactions achieved binding affinities below − 5 kcal/mol, indicating favorable binding stability (Fig. 3F). Quercetin, β-sitosterol, kaempferol, isorhamnetin, and wogonin were among the 14 exemplary active compounds found by integrative analysis of target overlap and docking performance (Fig. 3H).

To characterize intra-herb chemical diversity, hierarchical clustering and K-means clustering were applied to 32 herbs containing ≥ 3 active compounds. High compactness was demonstrated by the clustering results (mean Dunn index = 0.901). For downstream GCN-based synergy prediction, 24 core herbs were kept using an average silhouette score ≥ 0.45 as the selection criterion (Fig. 3I–J).

### GCN-predicted synergistic herbal formulas for AS

**Fig. 4:**
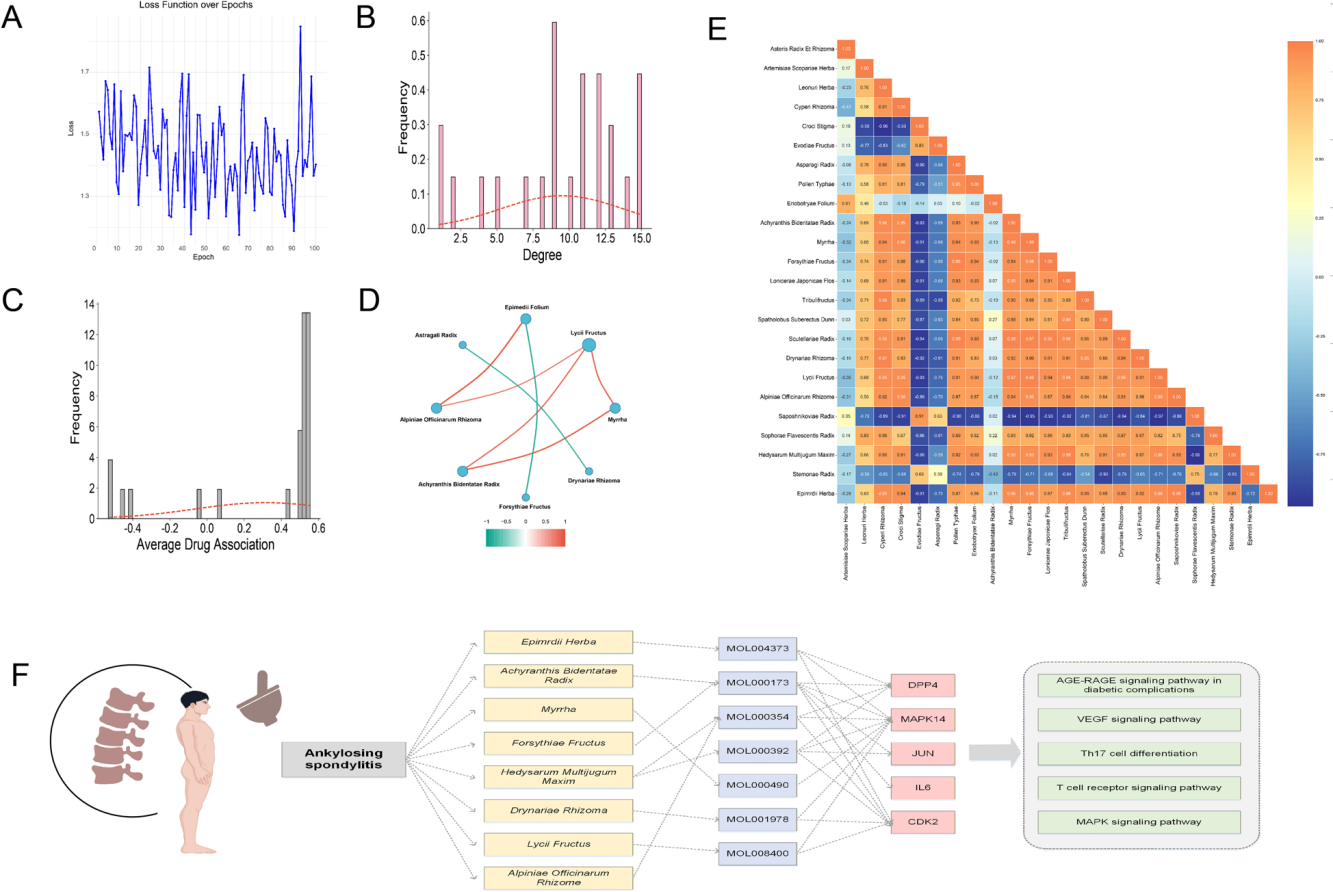
GCN-based TCM formula prediction and evaluation. **A** The training loss curve shows model convergence during parameter optimization. **B** GCN-derived node degree distribution. **C** Average drug association score distribution. **D** Predicted herbal formula Pearson correlation. **E** SVD decomposition heatmap showing inter-herb relationships. **F** The multilayer network shows herb, bioactive chemical, and disease-relevant target connections

To predict TCM combinations with therapeutic potential for AS, a GCN was used at the prescription level. The final model settings used in the reported experiments were as follows: hidden dimension = 32, learning rate = 1 × 10⁻^4^, dropout rate = 0.5, optimizer = Adam, loss function = binary cross-entropy, and maximum training epochs = 100. Gradient clipping was applied with a maximum norm of 1.0 to improve training stability. With slight oscillations probably caused by data heterogeneity and stochastic gradient updates, the training loss steadily reduced and plateaued with time, suggesting steady convergence (Fig. 4A). Out of the 225 projected herbal candidates produced by the trained model, 146 key herb pairs showed possible synergistic interactions. *Myrrha*, *Drynariae Rhizoma*, *Lycii Fructus*, *Epimedii Folium*, *Achyranthis Bidentatae Radix*, *Alpiniae Officinarum Rhizoma*, *Forsythiae Fructus*, *Astragali Radix,* were ranked using thresholds of GCN degree > 10 and average drug association > 0.5 by combining GCN topological features and SVD-based association scores ((Fig. 4B, C, Table 4).

**Table 4:** Core indicators of the ASD-A herbal prescription

Latin name of the herbs	Zd	dab	Overlap rate	Hscore	Mscore	Ec score	Ave silhouette score	GCN Degree	Average drug association
*Epimedii Folium*	− 40.702	1.863	0.005	0.019	18.123	0.016	0.450	13.000	0.526
*Achyranthis Bidentatae Radix*	**− **83.249	1.798	0.005	0.001	19.997	0.021	0.620	12.000	0.545
*Myrrha*	**− **69.121	1.822	0.005	0.003	19.096	0.009	0.530	15.000	0.536
*Forsythiae Fructus*	**− **43.717	1.841	0.005	0.006	27.199	0.023	0.510	11.000	0.535
*Astragali Radix*	**− **77.270	1.828	0.005	0.003	27.520	0.021	0.470	11.000	0.529
*Drynariae Rhizoma*	**− **44.353	1.850	0.006	0.005	11.734	0.033	0.550	15.000	0.533
*Lycii Fructus*	**− **56.767	1.832	0.005	0.002	26.304	0.006	0.710	13.000	0.531
*Alpiniae Officinarum Rhizoma*	**− **104.606	1.814	0.005	0.003	5.856	0.026	0.640	12.000	0.514

Pairwise synergy analysis further highlighted strong associations among *Myrrha*-*Achyranthis Bidentatae Radix* (P = 0.867), *Myrrha*-*Lycii Fructus* (P = 0.835), and *Achyranthis Bidentatae Radix*-*Lycii Fructus* (P = 0.807), suggesting shared pharmacological characteristics at both the compound and target levels and a central role in the core herbs (Fig. 4D). Nonetheless, the reliability of synergy estimation may be influenced by the completeness of compound annotation and the accuracy of target prediction models.

### Construction of a multilayer network and identification of key compounds

Network analysis demonstrated that the chosen traditional Chinese remedies are enriched with numerous bioactive compounds, with more than half of the herbs containing typical constituents such as quercetin, sitosterol, kaempferol, stigmasterol, luteolin, wogonin, and BA. Notably, these drugs target MAPK14 and affect many key signaling pathways, offering mechanistic insights into the molecular basis of ankylosing spondylitis (Fig. 4F).

### Comparative performance and ablation analysis

**Fig. 5:**
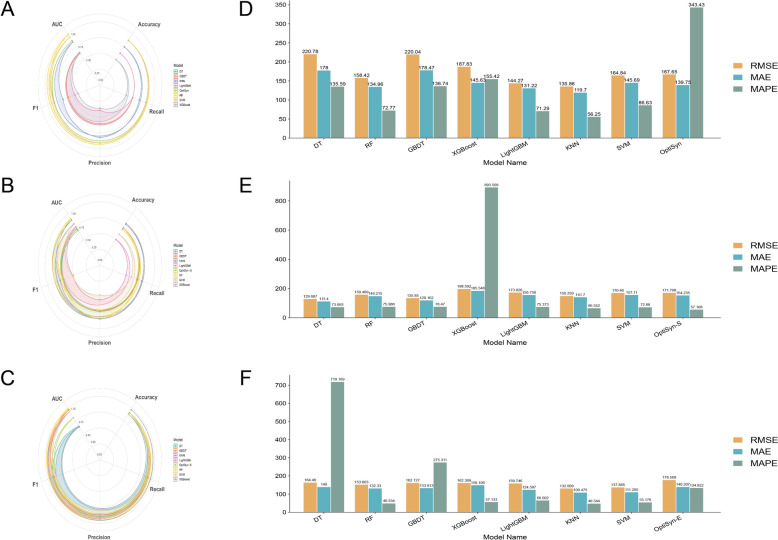
Model performance comparison and ablation analysis. **A**–**C** Classification metrics for OptiSyn, OptiSyn-S, and OptiSyn-E, respectively. **D**–**F** Regression metrics for OptiSyn, OptiSyn-S, and OptiSyn-E

OptiSyn outperformed other machine learning frameworks such as DT, RF, GBDT, SVM, XGBoost, LightGBM, and KNN (Fig. 5). For classification tasks, it outperformed traditional models in terms of accuracy, recall, precision, and F1 score (Fig. 5A). While its regression metrics were not globally minimum, combining drug-specific features with clinical usage data resulted in solid and sustained predictive performance, demonstrating good overall generalizability (Fig. 5D). These findings show that OptiSyn is a useful paradigm for modeling multi-target therapies in complicated disorders because it captures both phenotypic pharmacological properties and real-world application situations.

To investigate the contributions of critical determinants, an ablation analysis focused on compound similarity clustering and empirical clinical usage. The removal of compound clustering information (OptiSyn-S) resulted in herbal formula with high predictive performance; although classification and regression metrics decreased slightly relative to the full model, they remained superior to most baseline approaches (Fig. 5B, E; Table 5). Further elimination of clinical usage data (OptiSyn-E) necessitated adjustment of selection thresholds due to usually lower drug connectivity, resulting in new candidate formula (Table 6). Despite a slight decrease in AUC and RMSE compared to the entire model, accuracy, recall, precision, F1, and MAE improved, indicating that acceptable predictive performance may still be achieved with reduced empirical knowledge (Fig. 5C, F). Overall, the ablation experiments highlight the complementary value of compound structural information, drug connectivity, and clinical experience in model predictions, which is consistent with the utility of multi-source data integration in intelligent TCM formula screening and individualized intervention design.

**Table 5:** Core indicators of the ASD-B herbal prescription

Latin names of herbs	Zd	dab	Overlap rate	Hscore	Mscore	Ec score	GCN Degree	Average drug association
*Scutellariae Radix*	**− **28.442	1.836	0.008	0.008	34.073	0.011	15.000	0.527
*Achyranthis Bidentatae Radix*	**− **83.249	1.798	0.005	0.001	19.997	0.021	23.000	0.522
*Drynariae Rhizoma*	**− **44.353	1.850	0.006	0.005	11.734	0.033	15.000	0.523
*Forsythiae Fructus*	**− **43.717	1.841	0.005	0.006	27.199	0.023	18.000	0.516
*Sophorae Flavescentis Radix*	**− **73.068	1.814	0.005	0.009	9.875	0.015	18.000	0.502
*Astragali Radix*	**− **77.270	1.828	0.005	0.003	27.520	0.021	16.000	0.512
*Epimedii Folium*	**− **40.702	1.863	0.005	0.019	18.123	0.016	17.000	0.510
*Aquilariae Lignum Resinatum*	**− **70.299	1.811	0.006	0.005	11.160	0.021	22.000	0.513
*Bupleuri Radix*	**− **83.677	1.797	0.005	0.002	17.969	0.022	16.000	0.522

**Table 6:** Core indicators of the ASD-C herbal prescription

Latin names of herbs	Zd	dab	Overlap rate	Hscore	Ec score	Ave silhouette score	GCN Degree	Average drug association
*llicis Cornutae Folium*	**− **75.083	1.807	0.005	0.001	0.054	0.660	14.000	0.139
*Sarcandrae Herba*	**− **64.188	1.816	0.006	0.001	0.021	0.570	13.000	0.122
*Artemisiae Scopariae Herba*	**− **61.799	1.806	0.006	0.005	0.016	0.570	15.000	0.113
*lnulae Flos*	**− **59.843	1.800	0.005	0.004	0.022	0.620	12.000	0.111

### Functional enrichment and immune infiltration analysis

**Fig. 6:**
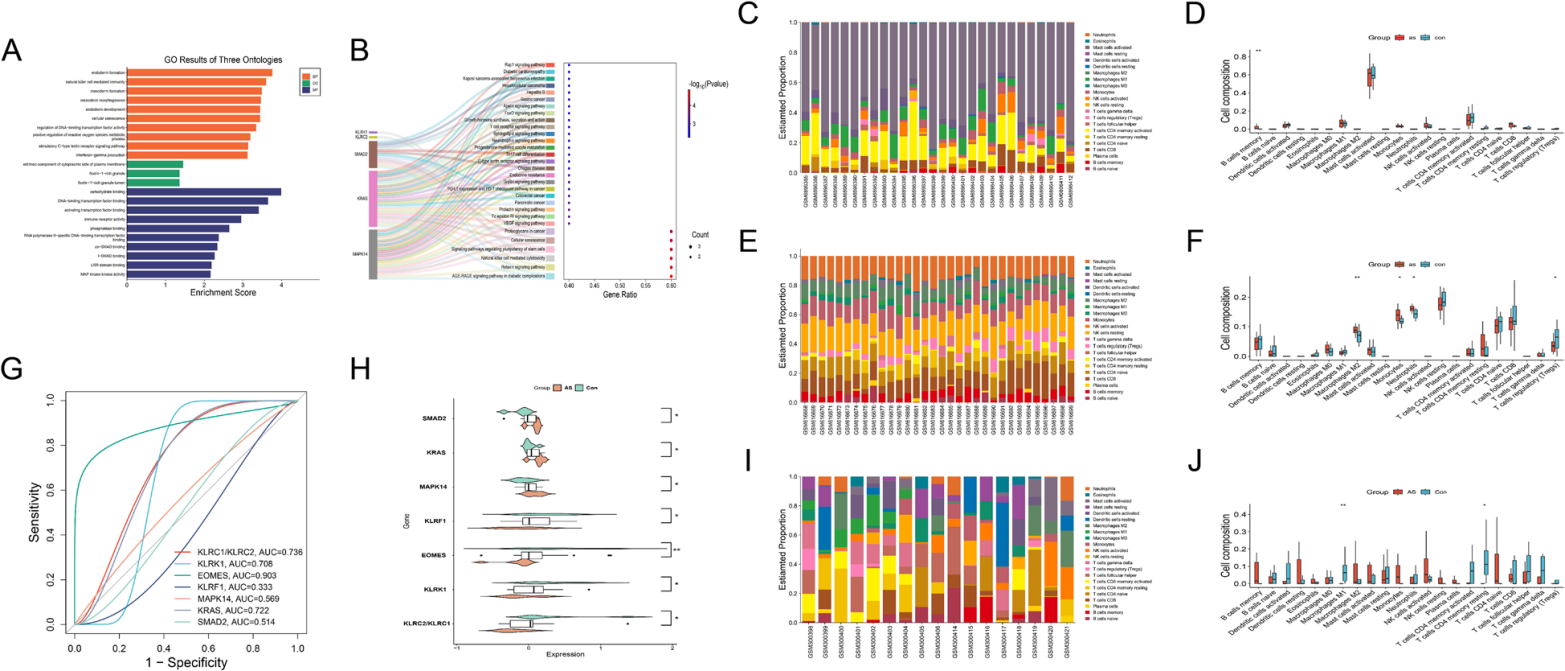
Multi-dimensional hub gene-disease analysis. **A** GO enrichment. **B** KEGG pathway enrichment. **C** GSE221786 of immune infiltration and **D** differential immune cell abundance. **E** GSE25101 of immune infiltration and **F** differential immune cell abundance. **G** Clinical diagnostic ROC analysis. **H** Hub gene validation. **I** GSE11886 of immune infiltration and (**J**) differential immune cell abundance

Hub genes are mostly implicated in oxidative stress, cellular senescence, immunological activation, chemotaxis, proliferation, differentiation, and protein complex dynamics, according to GO analysis (Fig. 6A). AGE-RAGE, FoxO, MAPK, VEGF, neurotrophin, and T-cell receptor signaling were highlighted by KEGG enrichment and were associated with metabolic, inflammatory, atherosclerosis, and cancer (Fig. 6B). Immune profiling showed considerable changes in M2 macrophage polarization and T-cell regulatory activity, as well as significant increases in B cells, macrophages, monocytes, and neutrophils combined with decreased T-cell proportions in AS (*P* < 0.05, Fig. 6C–F).

### Clinical diagnostic evaluation

To determine the diagnostic potential of the discovered hub genes, ROC curves were created using GEO datasets, and the corresponding AUC values were determined. EOMES had the highest diagnostic performance (AUC = 0.903), whereas KLRF1 had the lowest (AUC = 0.333). KLRC1, KLRC2, KLRK1, and KRAS all above 0.6 (Fig. 6G). Expression profiling showed significant upregulation of SMAD2, KRAS, and MAPK14 and downregulation of KLRF1, KLRC2, KLRK1, EOMES, and KLRC1 in AS samples (*P* < 0.05, Fig. 6H). Immune infiltration analysis highlighted marked differences in macrophage polarization and T-cell regulation (*P* < 0.05, Fig. 6I–J), supporting the strong immune relevance of these hubs. Overall, these genes exhibit robust predictive value as potential biomarkers for AS.

### Pharmacological interventions attenuate arthritis progression and restore spinal microarchitecture in AS mice

**Fig. 7:**
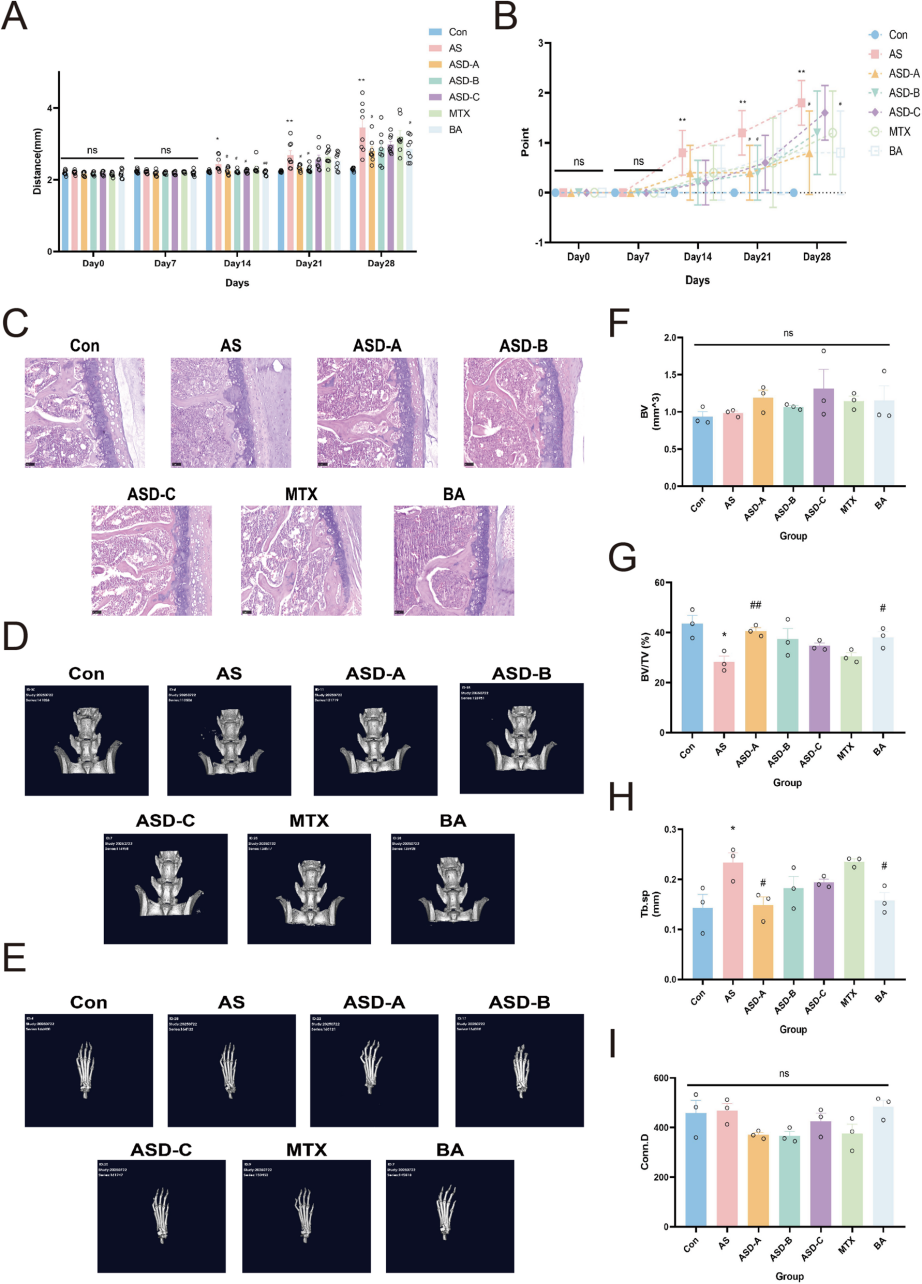
Therapeutic effects of different interventions on spinal and peripheral joint damage in AS mice. **A** Temporal changes in hind paw thickness across treatment groups (n = 8). **B** Arthritis swelling scores over time (n = 5). **C** Representative H&E-stained spinal sections (× 200) showing inflammation and tissue alterations (n = 3). **D** Three-dimensional micro-CT reconstruction of L5–L6 vertebrae and (**E**) left hind paw (n = 3). **F** Quantitative analysis of bone volume (BV), (**G**) BV/TV, (**H**) Tb.Sp, and (**I**) Conn.D. *P < 0.05 vs. Con; **P < 0.01 vs. Con; #*P* < 0.05 vs. AS; ##*P* < 0.01 vs. AS

Figure 7A, B shows that AS mice had significantly higher hind paw thickness and arthritis ratings than controls on day 14 post-induction (*P* < 0.05). Treatment with ASD-A, ASD-B, ASD-C, or BA significantly reduced paw edema by day 14 (*P* < 0.05). By day 28, there were no discernible differences between the ASD and MTX groups (*P* > 0.05), however ASD-A and BA treatments further reduced paw edema and histopathological arthritis scores in comparison to AS mice (*P* < 0.05). Histological investigation revealed significant cartilage disintegration, inflammatory cell infiltration, and fibroblast-like cell accumulation in AS spinal tissues compared to controls, all of which were reduced to variable degrees by all therapies (Fig. 7C).

Micro-CT analysis of L5–L6 vertebrae demonstrated severe trabecular deterioration in AS mice (*P* < 0.05), including reduced bone volume fraction (BV/TV), decreased trabecular number (Tb.N), and increased trabecular separation (Tb.Sp). All treatment groups partially restored these parameters (*P* < 0.05), with increases in BV/TV, decreases in Tb.Sp, and partial recovery of trabecular number and alignment. Connectivity density (Conn.Dn) showed no significant differences among groups (*P* > 0.05), though treated mice trended toward improvement relative to AS mice. No significant differences were observed in left paw assessments (*P* > 0.05). Collectively, these results indicate that AS induces severe bone microstructure disruption, which can be partially reversed by pharmacological intervention (Fig. 7D–I).

### Flow cytometry analysis of CD80/CD86 expression

**Fig. 8:**
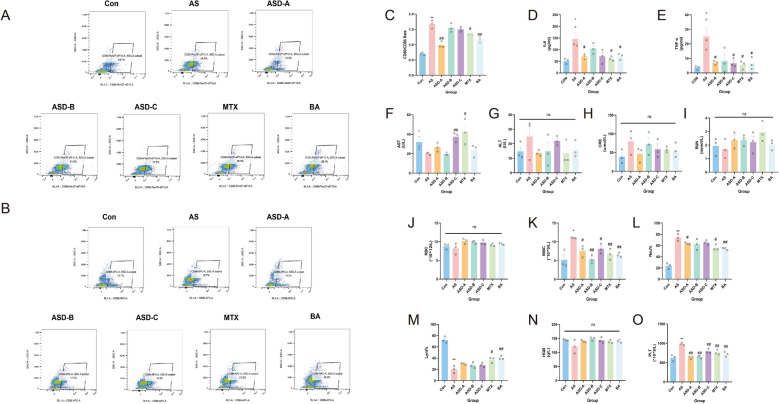
Effects of pharmacological interventions on immune activation, inflammatory responses, and safety profiles in AS mice. Flow cytometric analyses showing the gating strategy used to identify lymphocytes, exclude doublets, select live cells, and quantify **A** CD80⁺ and (**B**) CD86⁺ cells within the parent population. **C** Quantitative analysis of the CD80/CD86 ratio across groups. Serum inflammatory cytokine levels of **D** IL-6 and (**E**) TNF-α were quantified by ELISA. Safety assessments include liver function markers (**F**) aspartate aminotransferase and (**G**) alanine aminotransferase, renal function indices (**H**) creatinine and (**I**) urea nitrogen, and hematological parameters: (**J**) red blood cells, (**K**) white blood cells, (**L**) neutrophil proportion, (**M**) lymphocyte proportion, (**N**) hemoglobin, and (**O**) platelet counts (n = 3). *P < 0.05, **P < 0.01 vs. Con; #*P* < 0.05, ##*P* < 0.01 vs. AS

Flow cytometry showed that AS mice had increased CD80 expression and decreased CD86 expression, resulting in a significantly higher CD80/CD86 ratio (*P* < 0.01), indicating increased immunological activation. Drug interventions decreased the CD80/CD86 ratio to varied degrees, with ASD-A, MTX, and BA groups showing more dramatic drops (*P* < 0.05), approaching or restoring values comparable to Con. ASD-B and ASD-C exhibited no significant difference from the AS group (*P* > 0.05). Representative gating plots and compensation controls are shown in Supplementary Figure S1. These results suggest that AS causes an imbalance in co-stimulatory immune molecules, which may be partially addressed by specific therapies and help alleviate the disease (Fig. 8A–C).

### ELISA-based inflammatory and safety assessments

Following treatment of the condition, serum levels of TNF-α and IL-6 were measured. The AS group had considerably higher levels of both cytokines than the Con group (*P* < 0.05). TNF-α and IL-6 levels were significantly lowered (*P* < 0.05) by treatment with ASD-A, MTX, and BA, but ASD-B displayed a declining trend that was not statistically significant. TNF-α was dramatically reduced by ASD-C (*P* < 0.05), however IL-6 was not significantly affected. According to these findings, ASD-A, MTX, and BA successfully reduce inflammatory reactions in AS mice. All values remained within normal ranges, and the safety evaluation based on hematological parameters and hepatic and renal function indices revealed no significant anomalies between groups (Fig. 8F–O, P > 0.05).

### Optimization of drug concentration and exposure time

**Fig. 9:**
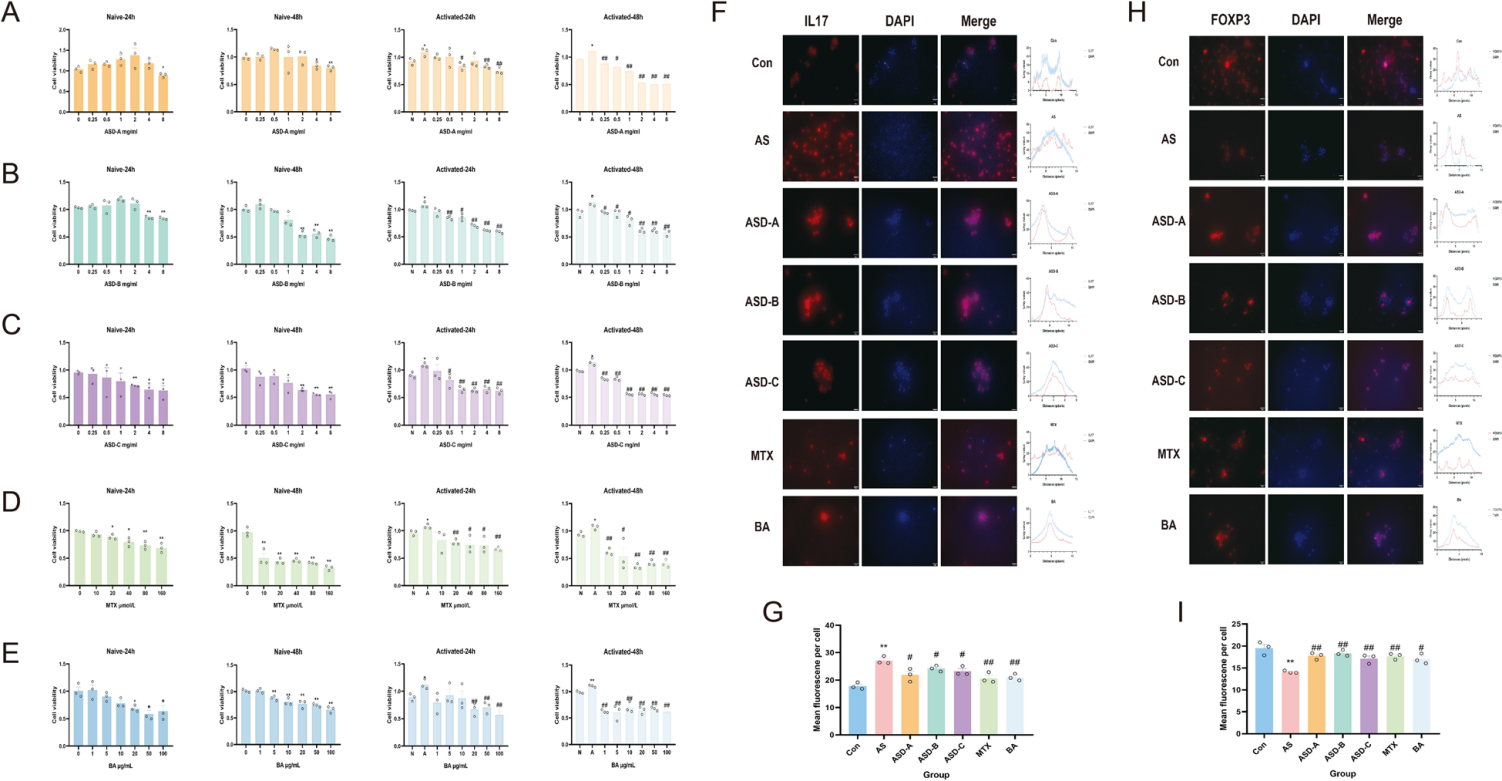
Optimization of drug treatment conditions and immunofluorescence validation in Jurkat cells. Dose-time response analyses were performed to determine the optimal concentrations and exposure durations of (**A**) ASD-A, (**B**) ASD-B, (**C**) ASD-C, (**D**) MTX, and (**E**) BA in Jurkat cells. **F**–**H** Immunofluorescence staining illustrates the effects of different treatments on IL17 and Foxp3 expression, with corresponding quantitative fluorescence analyses shown in (**G**) and (**I**) (n = 3). *P < 0.05, **P < 0.01 vs. Con; #*P* < 0.05, ##*P* < 0.01 vs. AS

To determine optimal intervention conditions, Jurkat cells were treated with graded concentrations of ASD-A, ASD-B, and ASD-C (0–16 mg/mL) for 24 or 48 h. Cell viability decreased in a dose-dependent manner. At 24 h, moderate viability reduction was observed with increasing concentrations; significant differences versus activated controls emerged at 1 mg/mL for ASD-A and 0.5 mg/mL for ASD-B and ASD-C (*P* < 0.05). At 48 h, viability declined more markedly across concentrations, with significant inhibition evident at 0.25 mg/mL for all three formulations (*P* < 0.05). Based on efficacy and tolerability, ASD-A (1 mg/mL) and ASD-B/ASD-C (0.5 mg/mL) for 24 h were selected for subsequent experiments (Fig. 9A–C). For MTX and BA, Jurkat cells were exposed to MTX (0–160 μM) or BA (0–100 μg/mL) for 24 or 48 h. Both agents exhibited clear dose- and time-dependent cytotoxicity (*P* < 0.05). Significant inhibition was observed at 24 h with MTX 20 μM and BA 20 μg/mL, whereas 48 h treatment induced marked suppression even at the lowest doses. Accordingly, MTX 20 μM and BA 20 μg/mL for 24 h were chosen as optimal conditions (Fig. 9D, E).

### Effects of different treatments on IL17 and Foxp3 expression in jurkat cells

The expression and subcellular localization of Foxp3 and IL17 in Jurkat cells were evaluated using immunofluorescence labeling (Fig. 9F–I). Compared with the Con, AS stimulation significantly increased IL17 fluorescence intensity and decreased Foxp3 expression (*P* < 0.05), indicating successful inflammatory activation. In contrast, compared with the AS-stimulated group, treatment with ASD formulations, MTX, or BA reduced IL17 fluorescence while increasing Foxp3 fluorescence (*P* < 0.05). Among the tested herbal formulations, ASD-A showed the strongest regulatory effect. Quantitative fluorescence analysis was consistent with the representative imaging results, confirming that these treatments modulated the IL17/Foxp3 axis.

### Selection and deconstruction of the optimal herbal formula

**Fig. 10:**
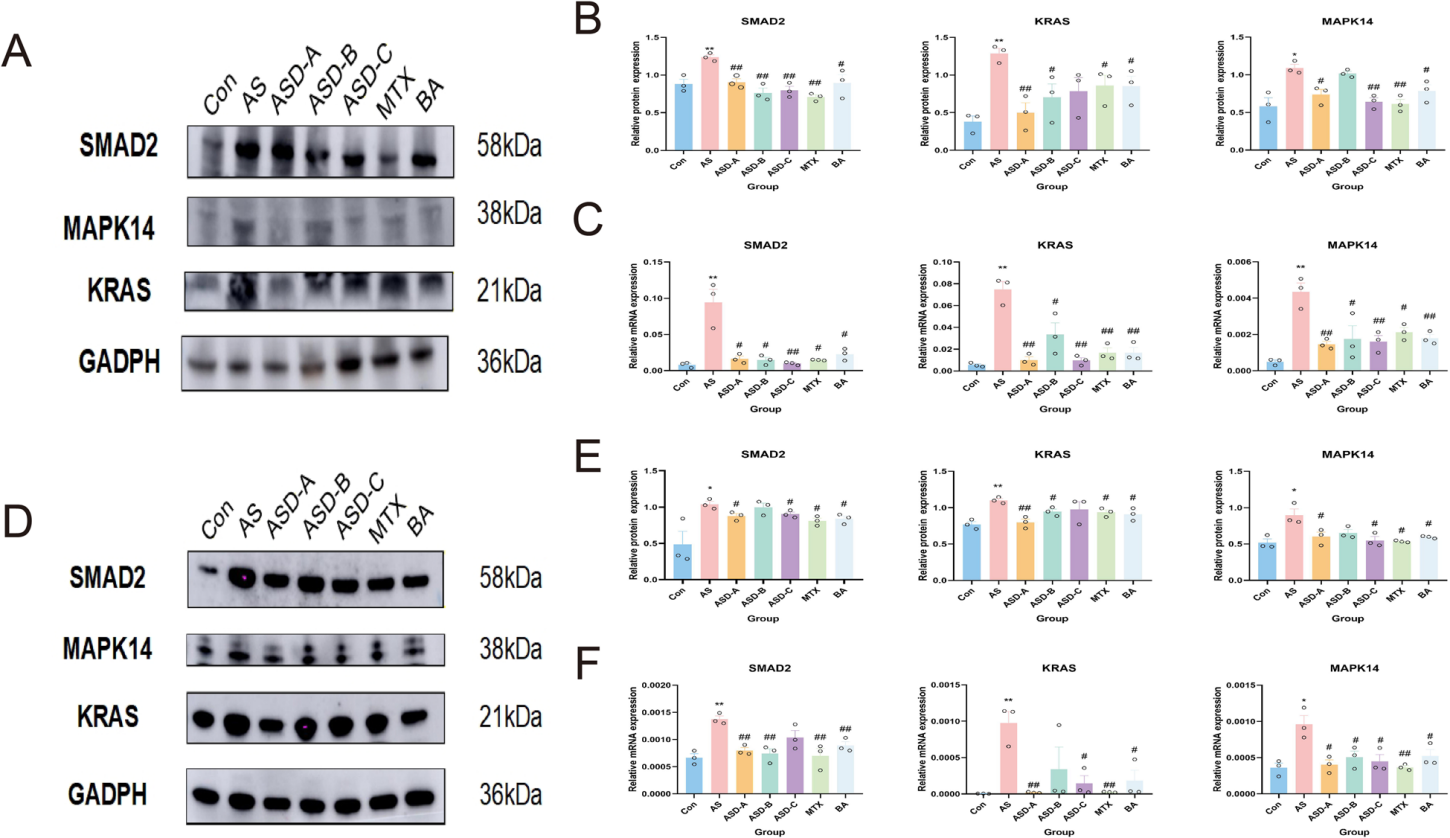
In vivo and in vitro validation of drug-mediated regulation of aberrant immune inflammation. Representative results show that different pharmacological interventions modulate abnormal immune responses by regulating hub genes in AS models (n = 3). In mice, drug treatments significantly attenuated excessive inflammatory activation through downregulation of SMAD2, KRAS, and MAPK14 at both (**A**, **B**) the protein and mRNA levels, as quantified by (**C**) qPCR. In CD3/CD28-stimulated Jurkat cells, pharmacological interventions suppressed aberrant immune activation via modulation of the same hub genes at (**D**, **E**) the protein and (**F**) transcriptional levels. *P < 0.05 vs. Con; **P < 0.01 vs. Con; #*P* < 0.05 vs. AS; ##*P* < 0.01 vs. AS

As depicted in Fig. 10, Western blot and qPCR analyses revealed increased immune-inflammatory activity in AS mice, with notable upregulation of KRAS, SMAD2, and MAPK14 at both the protein and mRNA levels compared to controls (*P* < 0.05). The expression of these hub proteins was significantly decreased by pharmacological intervention, with ASD-A exhibiting the strongest inhibitory effects. Consistently, CD3/CD28 co-stimulation of T cells induced robust inflammatory activation, reflected by elevated SMAD2, KRAS, and MAPK14 expression (*P* < 0.05), which was significantly attenuated by ASD-A, MTX, and BA (*P* < 0.05). Target-selective activities among various formulations were demonstrated by ASD-B's modest suppression of SMAD2 and MAPK14 and ASD-C's negligible effects on KRAS. These findings demonstrate the resilience of the suggested model and support ASD-A as the optimal formulation, which encourages further analysis.

**Fig. 11:**
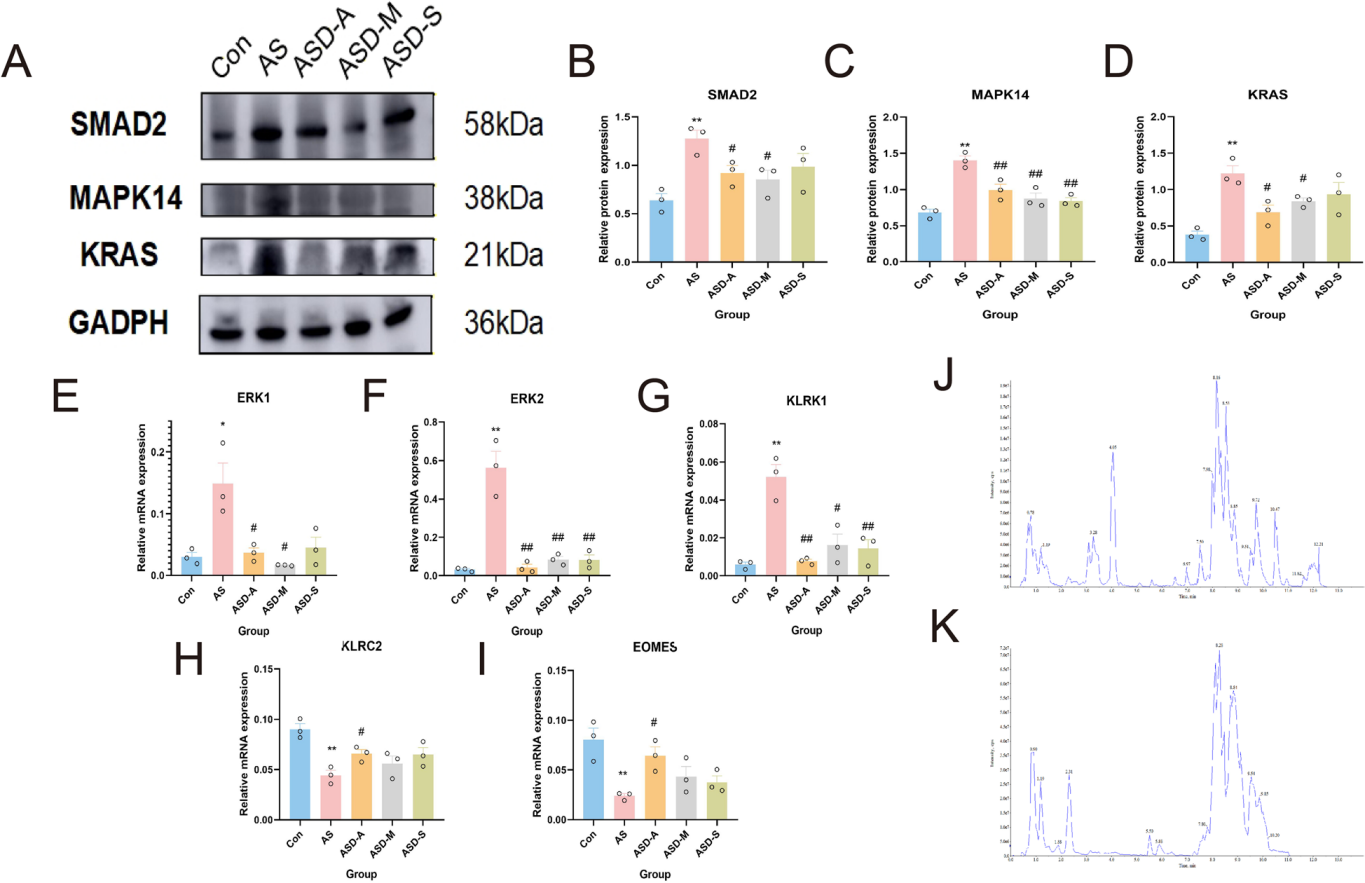
Deconstruction and validation of the ASD-A formula. The ASD-A formulation was deconstructed and experimentally validated to elucidate its anti-inflammatory mechanism. **A**–**D** Western blot analyses demonstrate that ASD-A and its derived sub-formulas attenuate excessive inflammation in AS mice by modulating SMAD2, MAPK14, and KRAS protein expression. **E**–**I** qPCR further quantified transcriptional changes in downstream immune-related genes, including ERK1, ERK2, KLRK1, KLRC2, and EOMES across treatment groups. **J**–**K** The major chemical constituents of ASD-A were identified by UPLC-Q-TOF–MS/MS. *P < 0.05 vs. Con; **P < 0.01 vs. Con; ^#^*P* < 0.05 vs. AS; ^##^*P* < 0.01 vs. AS

As illustrated in Fig. 11, ASD-A is composed of a variety of chemical categories, including flavonoids, phenolic acids, terpenoids, quinones, alkaloids, organic acids, steroids, and lipids, according to UPLC–Q-TOF–MS/MS profiling (Supplementary Table S1). Formulation dissection showed that while only ASD-M successfully downregulated SMAD2 and MAPK14 (*P* < 0.05), both the ASD-M and the ASD-S dramatically decreased KRAS protein levels (*P* < 0.05). Additionally, q-PCR revealed that ASD-A upregulated KLRC2 and EOMES while strongly suppressing ERK1, ERK2, and KLRK1 (*P* < 0.05). While ASD-S showed poor regulatory capacity, ASD-M partially recapitulated these effects but showed lower control of KLRC2 and EOMES. Based on the holistic and synergistic principles of traditional formula design, these results together show that the core components maintain significant bioactivity while the entire formula achieves superior and more coordinated modulation of hub pathways.

## Discussion

Lumbosacral discomfort and stiffness, spinal deformity, and limited mobility are typical symptoms of AS, a chronic inflammatory illness that mainly affects the sacroiliac joints, spinal entheses, paraspinal soft tissues, and peripheral joints [26]. A growing body of research suggests that oxidative stress, immunological dysregulation, inflammation, and autophagy-related pathways are important factors in the development and progression of AS [27, 28]. Even while the pharmacotherapies that are now on the market can reduce symptoms, side effects such gastrointestinal bleeding, gastrointestinal toxicity, and nephrotoxicity frequently limit their long-term usage. TCM has unique therapeutic potential due to its multi-component formulations and holistic treatment philosophy. A revolutionary paradigm has been brought about by the incorporation of artificial intelligence into drug discovery, allowing for faster target-compound mapping and offering a new computational framework for the logical development and improvement of TCM formulae [29].

In order to capture intricate relationships between herbal components, hub genes, and multi-dimensional scoring metrics, we created a multilayer network modeling and prediction framework in this study that combines network topology features, target overlap, molecular virtualization, and synergistic clustering–based filtering. With this framework, herbal combinations can be screened without relying on explicit dose–response assumptions [12]. Notably, it offers a methodical measurement of the potential and significance of inter-formula interactions, which is in close accord with the conventional TCM theory of *Jun-Chen-Zuo-Shi* principle. Differential expression analysis, random walk algorithms, dimensionality reduction, Mendelian randomization, and module partitioning were used to identify eight candidate AS-associated hub genes from single-cell transcriptomic data, which may help characterise immune complexity and prioritise potential intervention targets. KRAS, KLRC2, KLRC1, KLRK1, and KLRF1 were found to be prioritized AS-associated genes, mostly expressed in NK and T cells, and have been implicated in TCR signaling regulation in CD8⁺ T-cell subsets, antigen-driven immune activation, inflammation, and cell proliferation [30–32]. Simultaneously, RNA-seq and immune infiltration investigations showed that AS pathogenesis is strongly linked to significant infiltration of monocytes, neutrophils, T cells, NK cells, and dendritic cells. SMAD2 functions as a critical intracellular signal transducer and transcriptional regulator, activating gene transcription via TRE elements upon formation of the SMAD2/SMAD4 complex downstream of TGF-β1 signaling [33]. From an immunopathological perspective, AS is characterized by impaired immune barrier integrity, including increased intestinal permeability and deficiencies in immunoglobulins and complement, which collectively promote excessive secretion of pro-inflammatory cytokines and chemokines such as IL17, MAPK14, CCL9, and TNF-α. These events drive aberrant activation of NF-κB, MAPK, and STAT pathways while suppressing Treg-mediated immunoregulation, ultimately disrupting T-cell immune homeostasis [34, 35]^.^

An ASD formulation enriched with *Epimedii Folium*, *Achyranthis Bidentatae Radix*, *Lycii Fructus*, and *Epimedii Herba* was found using the interpretable model. This formulation contained important bioactive ingredients such as kaempferol, quercetin, BA, curcumin, and calycosin. It has been demonstrated that kaempferol specifically inhibits PIM1 kinase, attenuates inflammatory arthritis, and improves Treg function by upregulating CTLA4, IL-10, and Foxp3 [36, 37]. Through coordinated modulation of immune-related genes and miRNAs, curcumin restores T-cell homeostasis in AS, according to randomized clinical data [38]. By inhibiting IL-17-driven pathways and pathogenic T-cell subsets, BA demonstrates strong anti-inflammatory and anti-fibrotic properties, whereas calycosin contributes immunomodulatory action through antioxidant and macrophage-polarizing mechanisms [39, 40]. The therapeutic potential of the improved formulation is supported by the plausible convergence of these drugs on AS-associated hub genes, which also calls for more mechanistic confirmation.

The study empirically confirmed the biological reliability of OptiSyn predictions. ASD formulations consistently influenced essential AS regulatory hubs, validating that the model-selected drug combinations effectively target crucial nodes of the disease. ASD-A treatment partially inhibited IL17-related inflammatory signaling and restored Foxp3 expression in activated Jurkat cells, suggesting a rebalancing of effector and regulatory T-cell responses. These results correspond with previous enrichment analyses identifying MAPK, T-cell receptor, and oxidative stress pathways, so building a cohesive prediction-mechanism-validation framework. Among candidate formulations, ASD-A showed superior regulatory efficacy across key genes and immune-inflammatory outcomes. Ablation analysis further demonstrated that incorporating compound structural similarity and clinical prescription priors improves the selection of formulations with stable biological activity. In addition, BA emerged as a potential active contributor, exhibiting strong MAPK14 binding and measurable anti-inflammatory effects, supporting its role as a core functional component and a promising small-molecule lead for AS. Notwithstanding these advantages, numerous constraints persist. Incomplete herbal datasets may lead to predictive bias, and mechanistic validation was largely restricted to essential gene-level investigations, with scant in vivo and clinical evidence. In the future will amalgamate multi-omics data to enhance sample representation, uncover new targets, and propel translational drug research forward.

## Conclusion

An interpretable multi-layer network-based methodology for finding drug combinations that combines molecular docking, target overlap, network topology, hub gene prioritization, multi-omics analysis, and structural similarity grouping. Regardless of dose–response assumptions, the framework allows for the methodical selection of the optimal therapeutic drug combinations. The robustness and translational potential of the suggested computational approach are demonstrated by the in vitro validation of the anticipated formulation in AS, which confirms its biological significance.

## Supplementary Information

**Additional file 1.**

**Additional file 2: Table S1. **The detailed information of identified components in positive ion mode and negative ion mode by UPLC-QTOF-MS.

## Data Availability

The source codes and the data of OptiSyn are available at https://github.com/viviiviiikikiikiii/OptiSyn.

## Abbreviations

AI: Artificial Intelligence
AS: Ankylosing Spondylitis
BA: Betulinic Acid
DEGs: Differentially Expressed Genes
ELISA: Enzyme-Linked Immunosorbent Assay
GCN: Graph Convolutional Network
GEO: Gene Expression Omnibus
GO: Gene Ontology
KEGG: Kyoto Encyclopedia of Genes and Genomes
MTX: Methotrexate
PPI: Protein–Protein Interaction
qPCR: Quantitative Polymerase Chain Reaction
ROC: Receiver Operating Characteristic
AUC: Area Under the Curve
scRNA-seq: Single-Cell RNA Sequencing
SMR: Summary-data-based Mendelian Randomization
SVD: Singular Value Decomposition
TCM: Traditional Chinese Medicine
TCMSP: Traditional Chinese Medicine Systems Pharmacology Database
WGCNA: Weighted Gene Co-expression Network Analysis
