# Supplementary figures and images for: OptiSyn: an interpretable, multi-omics–driven graph convolutional network framework for synergy-oriented drug combination design in disease treatment
Source: Chin Med. 2026 Mar 24;21:101. doi: 10.1186/s13020-026-01385-1 (PMC13011277; doi:10.1186/s13020-026-01385-1)

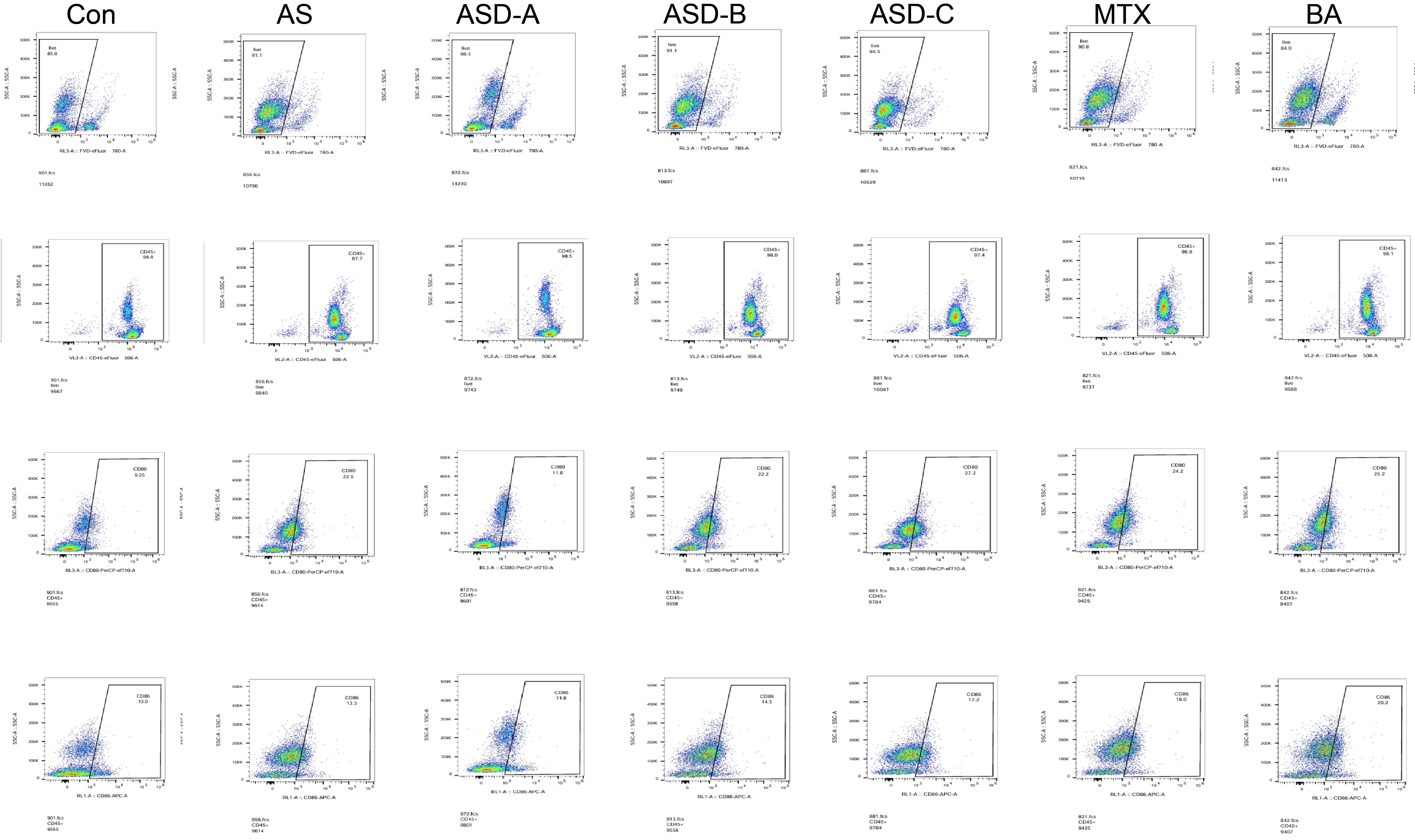

Supplement: Supplementary file 1 — Additional file 1. [file 13020_2026_1385_MOESM1_ESM.jpg]
